# Camel milk as an alternative treatment regimen for diabetes therapy

**DOI:** 10.1002/fsn3.2078

**Published:** 2021-02-02

**Authors:** Humaira Hussain, Feroza Hamid Wattoo, Muhammad Hamid Sarwar Wattoo, Muhammad Gulfraz, Tariq Masud, Imam Shah, Sakhawat Ali, Seyed Ebrahim Alavi

**Affiliations:** ^1^ University Institute of Biochemistry and Biotechnology PMAS ‐ Arid Agriculture University Rawalpindi Pakistan; ^2^ Department of Chemistry Pakistan Institute of Engineering and Applied Sciences Islamabad Pakistan; ^3^ National Veterinary Laboratories Islamabad Pakistan; ^4^ Department of Food Technology PMAS ‐ Arid Agriculture University Rawalpindi Pakistan; ^5^ Department of Pilot Nanobiotechnology Pasteur Institute of Iran Tehran Iran

**Keywords:** antidiabetic activity, glibenclamide, hepatoprotective effect, triglyceride

## Abstract

Camel milk is a valuable source of nutrition with a wide range of therapeutic effects. Its unique composition helps to regulate the blood glucose level. The current study is aimed to evaluate the antidiabetic and hepatoprotective effects, as well as lipid profile restoration of camel milk in the diabetic mouse model. This innovative study evaluates the therapeutic effects of camel milk in diabetic mice by simultaneous measurement of blood glucose, HbA1c, ALT, AST, TG, cholesterol, and histopathological studies. The results showed that camel milk has significantly reduced blood glucose, HbA1c (*p* < .001), aspartate transaminase (AST), alanine transaminase (ALT) (*p* < .01), triglyceride (TG), and cholesterol (*p* < .01), compared to that in the diabetic control group. Also, the therapeutic effects of camel milk were completely comparable with the antidiabetic drug glibenclamide. The results of this study suggest that camel milk could be used as a proper alternative treatment regimen for diabetes therapy.

## Graphical abstract

Camel milk as a natural safe product can be used as an alternative treatment regimen in diabetes therapy. Potency of camel milk is to restore the activity of hepatocyte enzymes such as ALT and AST. Camel milk is an effective approximately as much as glibenclamide to normalize blood TG and cholesterol concentrations.

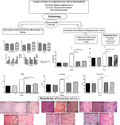

## 1 INTRODUCTION

Diabetes is a set of metabolic diseases recognized by high blood glucose levels arising from complications in insulin production, insulin use, or both (Alavi et al., 2020; Fan, 2017). Regarding the information obtained from International Diabetes Federation in 2015, nearly 415 million people suffered from diabetes worldwide, and this is expected to be more than 640 million by the year 2040 (Papatheodorou et al., 2018). Also, according to the National Diabetes Survey of Pakistan, 26.3% of adults (≥20) were diabetic in 2016–2017 that was equal to 27.4 million people (Basit et al., 2020). Diabetes is a complex and chronic disease that affects many organs of the body. It increases the risk of various disorders, such as retinopathy, neuropathy, nephropathy, and cardiovascular disease (Alavi, et al., 2019; Preguiça et al., 2020). These disorders are a remarkable cause of increased morbidity and mortality among diabetic people (Eid et al., 2019).

Milk plays a significant role in nutrition and health due to having a unique composition of proteins, vitamins, carbohydrates, and minerals. Camel milk is a valuable and common source of nutrition in many countries, in which it is different not only in composition but also in function (Alabdulkarim, 2012). It is a rich source of immunoglobulins (G and A), vitamins [A, B_2_, C (highest), and E], and minerals (Na, K, Fe, Cu, Zn, and Mg) while sugar, proteins, and cholesterol are not present in high concentration in camel milk (Hammam, 2019; Mullaicharam, 2014). Also, it contains less quantity of short‐chain fatty acid carotene and a high concentration of long‐chain fatty acids (Al‐Nasseri et al., 2019). Camel milk has high concentrations of antimicrobial agents. The concentration of lactoferrins in camel milk is about twofold to sixfold higher as compared to that in cow milk (Niaz et al., 2019). Comparing human and camel milk, both compositions are almost the same as they are free of β‐lactoglobulin, while α‐lactalbumin is the major protein in both (Merin et al., 2001). Also, as both are rich in β‐casein, camel milk can be digested easily without any allergic effect (Gizachew et al., 2014).

The therapeutic effects of camel milk are known in different parts of the world due to the presence of bioactive agents in the milk. It has been used for the treatment of different diseases, such as dropsy, jaundice, tuberculosis, kala‐azar (Ali & Al‐Attar, 2020) and anemia (Abdurahman, 2018). Furthermore, it has been demonstrated that camel milk is very effective in the treatment of liver disorders to normalize their functions (Darwish et al., 2012). Also, anticancer (Yang et al., 2019), antiallergic (Gizachew et al., 2014), and antidiabetic effects (Hammam, 2019) of camel milk have been proved. In addition, it is highly digestible with antioxidative capability (Ugwu et al., 2019). In different regions of Asia, the Middle East, and Africa, camel milk is used for the treatment of diabetes mellitus due to the therapeutic effect of its immunoglobulins on β cell of Langerhans islets (Agrawal et al., 2007).

The present study is aimed to evaluate the antidiabetic and hepatoprotective effects of camel milk in mice. In this regard, mouse diabetic model was established, and their serum glucose level, HbA1c, total cholesterol, and triglycerides were measured. Also, the histopathological studies were performed in the heart, liver, and pancreas.

## 2 MATERIALS AND METHODS

### 2.1 Chemicals

Streptozotocin (purchased from Sigma‐Aldrich), sodium citrate, glibenclamide, and ethylenediaminetetraacetic acid (EDTA) were of analytical reagent grade purity were used.

### 2.2 Animals

Healthy male and female mice were obtained from the National Veterinary Laboratory, Islamabad, Pakistan. They were kept in friendly house conditions suitable for animal survival with a temperature of 25 ± 2°C and humidity. Twelve‐hour light and dark cycles were provided. Animals had free access to “water ad libitum” and were provided with a standard animal diet. All animal experiments were approved by the ethics committee of PMAS‐Arid Agriculture University, Rawalpindi, Pakistan.

### 2.3 Collection and analysis of camel milk

Fresh camel milk was collected from the herd of camels on alternating days and was kept in cool and sealed containers for further analysis in the laboratory of University Institute of Biochemistry and Biotechnology, PMAS—Arid Agricultural University, Rawalpindi, Pakistan.

### 2.4 Evaluation of antidiabetic activity

#### 2.4.1 Experimental design

Sixty animals were divided into five groups, each having 12 animals (Ebaid et al., 2013).

Group 1: No treatment
Group 2: Camel milk
Group 3: Diabetic mice
Group 4: Diabetic mice + raw camel milk
Group 5: Diabetic mice + glibenclamide

#### 2.4.2 Experimental induction of diabetes

The animals in groups 3, 4, and 5 were induced diabetes after 24 hr fasting by a single injection of streptozotocin (40 mg/kg body weight) given intraperitoneally (Hu et al., 2017). Streptozotocin is a selective beta‐cell genotoxicant, and its single high dose administration induces a quick onset of diabetes by producing a sufficient amount of DNA adducts, leading to over activation of polyadenosine diphosphate ribose synthetase in the base excision repair pathway (Burns & Gold, 2007). Streptozotocin was prepared in 0.1 M citrate buffer (pH 4.5). The animals were fed 20% glucose solution after injection for one night to avoid hypoglycemic death of animals due to the injections. The animals which were given injections of Streptozotocin showed glycosuria that was determined by Benedict's test (Ramalingam et al., 2020). Diabetes was confirmed by measuring the concentration of blood glucose level 96 hr after drug administration. Animals having more than 240 mg/dl of blood glucose level were considered diabetic to be used in this study.

#### 2.4.3 Treatment

- Camel milk

The mice in groups 2 and 4 received fresh camel milk daily (83 ml/kg) for 7 weeks (Mansour et al., 2017). The mice were given free access to camel milk other than their regular feed. Camel milk was replaced in feeding bottles daily.

- Antidiabetic drug

The diabetic mice in group 5 were given antidiabetic drug glibenclamide to compare its effect with camel milk. The drug was prepared in distilled water and given in a dose of 600 µg/kg body weight to each mouse orally in the morning (Zangeneh et al., 2018).

### 2.5 Collection and processing of blood and tissue samples

After treatment for 7 weeks, the mice were etherized, and blood samples were taken from their heart in 2 tubes. In tube 1, the blood was centrifuged to obtain serum, and in tube 2, the blood was mixed with EDTA to obtain plasma. Next, mice were sacrificed, and their heart, liver, and pancreas were removed. The collected tissues were instantly preserved in 10% formalin for histopathological studies by using hematoxylin and eosin (H & E) staining (Ghaferi, Amari, et al., 2020). For this purpose, the successive sections of paraffin‐embedded tissues were prepared (Alavi, et al., 2019). The sections were then placed on glass slides, deparaffinized, rehydrated, and finally stained with H & E (Al Harthi et al., 2019; Ghaferi et al., 2020; Sale et al., 2020). Serum glucose level was quantified by using a reagent kit (Adaltis) according to the method described by Trinder (1969). Total cholesterol level and triglycerides (TG) were estimated (Aloud et al., 2018; Benkhaled et al., 2020) by using reagent kits (Chengxinde reagent company). HbA1c and liver enzymes (ALT and AST) were estimated and verified with kits (Ghaferi, Asadollahzadeh, et al., 2020; Lv et al., 2020) by using an HbA1c test kit (Beijing Wantai Dro Co., Ltd.) and AST and ALT commercial kits (Jiancheng Bioengineering Institute).

## 3 RESULTS AND DISCUSSION

In the present study, the efficacy of camel milk to treat diabetic mice was evaluated. For this purpose, diabetic animal model was established through the intraperitoneal injection of antibiotic streptozotocin.

The blood glucose evaluation confirmed that all mice got diabetes. Diabetes is a risk factor for the development of liver disorders, such as fibrosis and nonalcoholic fatty liver disease (Brouha et al., 2018). Therefore, in addition to blood glucose and HbA1c levels, serum concentrations of liver biomarkers, including ALT and AST, were measured. Also, dyslipidemia is a diabetes consequence characterized by hypertriglyceridemia and elevated LDL cholesterol (Lazarte & Hegele, 2020); therefore, serum TG and total cholesterol were measured.

**FIGURE 1:**
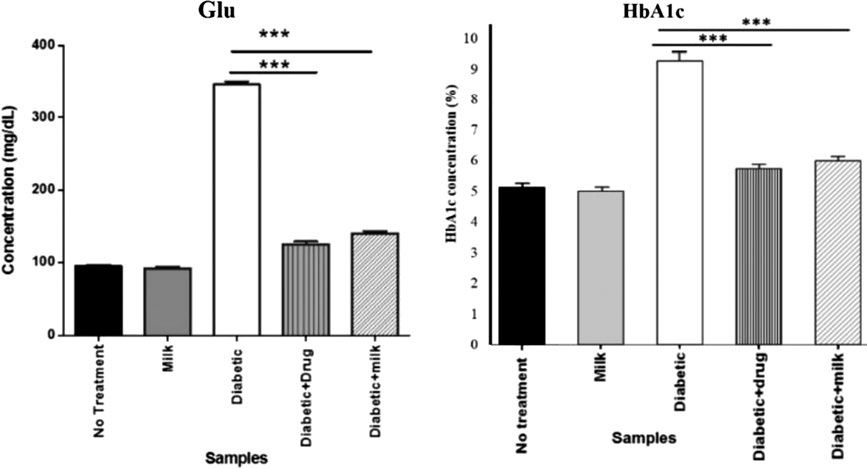
Mean blood glucose (Glu) and HbA1c concentrations in different groups of mice treated with the preparations of camel milk and glibenclamide

Figure 1 shows that the mean blood glucose in diabetic mice is 346 mg/dl, and this value in diabetic mice fed by camel milk is decreased to 140 mg/dl, which is not significantly different from the diabetic animal receiving glibenclamide (blood glucose of 125 mg/dl). Also, the pattern changes in HbA1c in different groups of mice are in accordance with the blood glucose levels. The results of the present study are in accordance with the above‐mentioned studies that camel milk statistically decreased the blood glucose and HbA1c concentrations in diabetic mice (*p* < .001).

A few studies evaluated the antidiabetic effects of camel milk (R. Agrawal et al., 2004; Hamad et al., 2011; Khan et al., 2013). Agrawal et al. (2004) evaluated the potency of camel milk to glycemic control in diabetic rats that diabetic animals which received camel milk had considerably lower blood glucose levels compared with the control diabetic animal. Hamad et al. (2011) investigated the antidiabetic effects of camel milk compared with cow and buffalo milk on diabetic Sprague‐Dawley rats proved that camel milk had higher hypoglycemic effects (49%) compared with cow and buffalo milk (11%).

Results of Table 1 confirmed the potency of camel milk to restore the activity of ALT and AST enzymes as associated with hepatocellular biomarkers. These protective effects of camel milk could result from its antioxidant activity and probable chelating effects on toxicants (Al‐Humaid et al., 2010). Hamad et al. (2011) and Khan et al. (2013), assessed hepatoprotective effects of camel milk in the diabetic animal model. Hamad et al. (2011) evaluated the hepatoprotective effects in diabetic rats and observed an improvement in the activities of ALT and AST by 41% and 38%, respectively, compared with the control group. Khan et al. (2013) assessed hepatoprotective effects in diabetic rats, and the results demonstrated that camel milk approximately restored the functionality of ALT (70 U/L) and AST (98 U/L) compared with the healthy control group (ALT and AST of 75 and 45 U/L, respectively).

**TABLE 1:** The effects of camel milk and the drug (glibenclamide) on the concentrations of ALT and AST in different groups of diabetic and nondiabetic mice

Groups	Biomarker	
	ALT (U/L)	AST (U/L)
No treatment	59.8 ± 1.15	91.0 ± 2.55
Milk	61.7 ± 0.76	93.4 ± 2.49
Diabetic	114 ± 3.83	144 ± 2.88
Diabetic + Drug	67.5 ± 3.39	103 ± 2.59
Diabetic + milk	77.8 ± 3.03	122 ± 5.59

**FIGURE 2:**
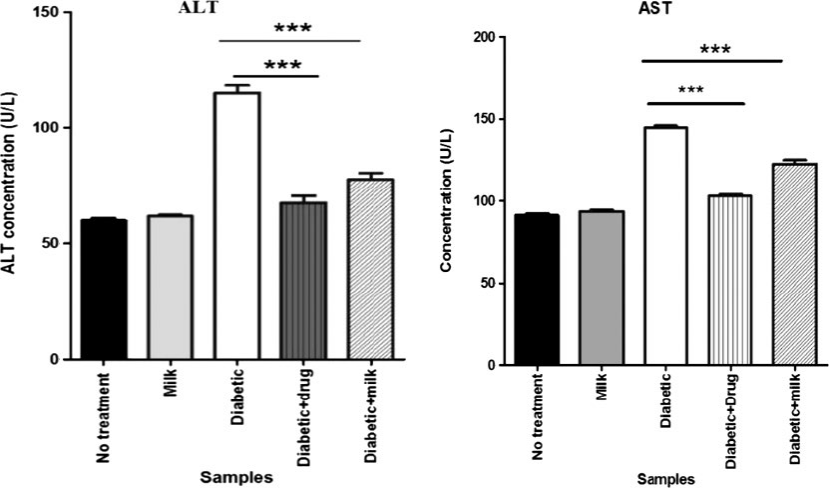
Mean ALT and AST concentrations in diabetic mice received camel milk and glibenclamide

The results of the current study (Figure 2) demonstrated that camel milk significantly restored the functionality of ALT and AST enzymes compared with the control diabetic mice (*p* < .001). While ALT and AST values in diabetic mice were 114 and 144 U/L, respectively. The mean values in diabetic mice after camel milk treatment were 78 and 122 U/L. Also, ALT and AST concentrations in diabetic mice treated with glibenclamide were 67 and 102 U/L, respectively, indicating the comparable efficacy of camel milk with glibenclamide in restoring the functionality of ALT and AST.

The high levels of vitamins (A, B_2_, and C, E act as an antioxidants) are present in camel milk which are useful in preventing tissue damage by toxic materials, such as streptozotocin (Sadek et al., 2016). Furthermore, camel milk contains a high level of zinc, a trace element required for living organisms (Sadek et al., 2016). There are more than 300 enzymes, which required Zn for their activity and a relationship with many body enzymes (Marreiro et al., 2017).

**FIGURE 3:**
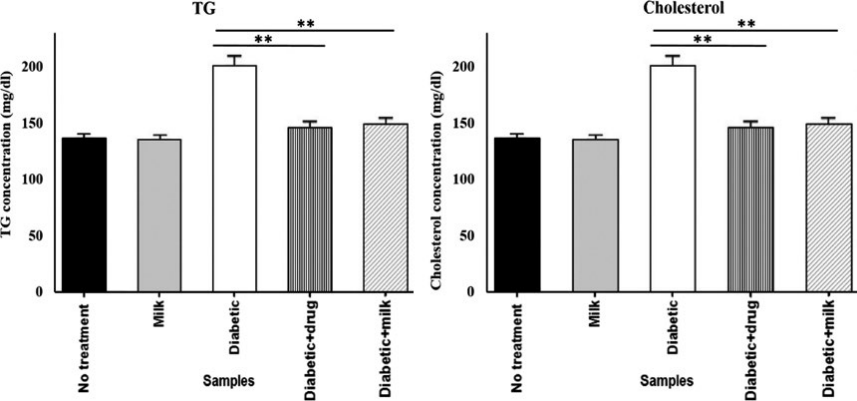
Mean TG and cholesterol concentrations in diabetic mice received camel milk and glibenclamide

The results of the present study (Figure 3) showed that camel milk was effective in reducing the blood TG and cholesterol concentrations by 26% and 22%, respectively, compared with the control diabetic mice (*p* < .01). More importantly, the efficacy of camel milk in the normalization of the blood TG and cholesterol in diabetic mice was comparable to the diabetic mice given antidiabetic drug glibenclamide. While the blood TG and cholesterol concentrations in diabetic mice which received camel milk were 149.8 and 186 mg/dl, respectively, the mean values in diabetic mice which received glibenclamide were 146 and 169 mg/dl, respectively. These findings support the idea that camel milk can be used as an alternative regimen for diabetes treatment, although various studies evaluated the antidiabetic effect of camel milk in in vivo environment (Agrawal et al., 2020; Aqib et al., 2019; Korish et al., 2020).

Khan et al. (2013) and Sboui et al. (2010) evaluated the therapeutic effects of camel milk on the lipid profile of diabetic animals. Khan et al. (2013) showed that camel milk restored the lipid profile to near control levels. The results demonstrated that camel milk decreased the blood cholesterol and TG concentrations by 34% and 35%, respectively, in diabetic rats. In addition, camel milk decreased the blood cholesterol concentration by 26% in diabetic dogs, with small decrease in blood TG concentration (Sboui et al., 2010).

The present study is the first report which evaluated the factors of blood glucose, HbA1c, total cholesterol, triglycerides, ALT, and AST simultaneously.

### 3.1 Histopathological findings

**FIGURE 4:**
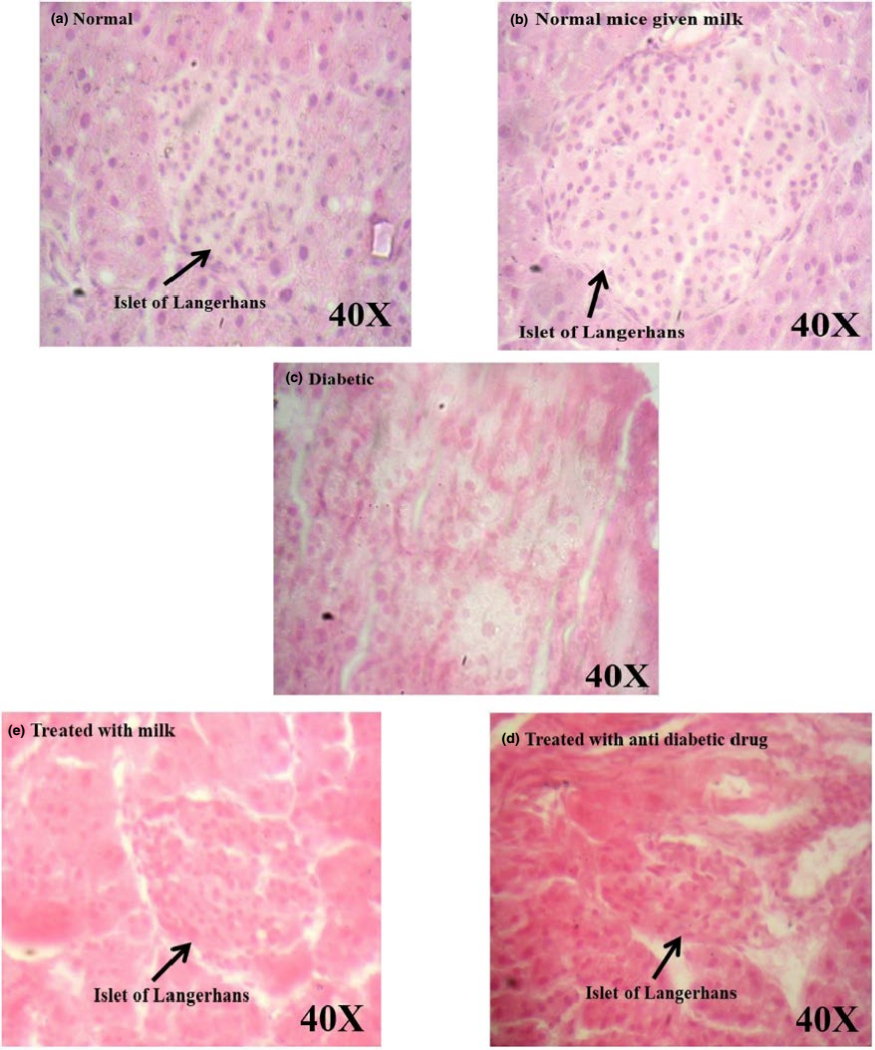
Histopathological evaluation of the pancreas by using H & E staining in different groups of mice. As the figure shows, there were no considerable pathological changes in the pancreases of diabetic mice received glibenclamide drug (d) and camel milk (e) and were significantly less than the pathological changes (size shrinkage, reducing the number of the islets, and beta‐cell destruction) observed in the pancreases of control diabetic mice (c)

Normal histological structure of the pancreas with normal‐sized islets (Figure 4) was observed in normal mice, while size shrinkage, decrease in the number of the islets, and destruction of beta cells were observed in control diabetic mice.

**FIGURE 5:**
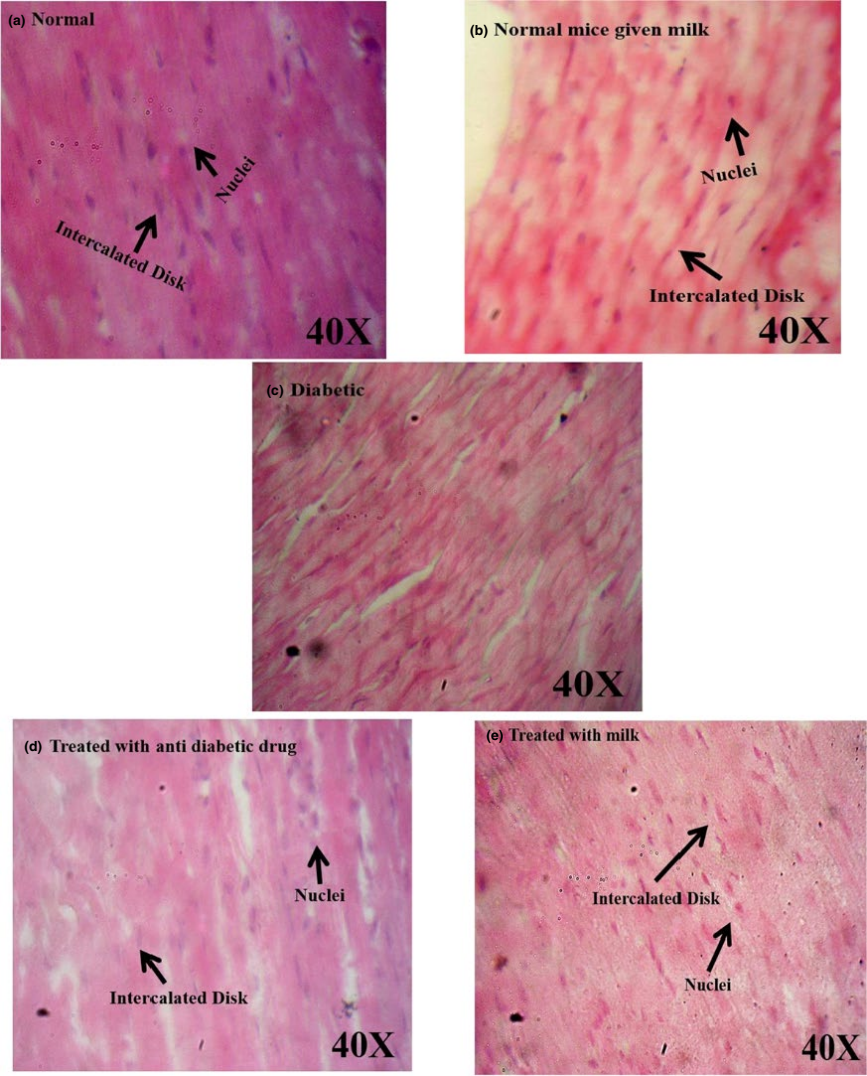
Histopathological evaluation of the heart in different groups of mice by using H & E staining. As the figure shows, there were no considerable pathological changes in the hearts of diabetic mice received glibenclamide drug (d) and camel milk (e) and were significantly less than the pathological changes (confused cellular nuclei and degenerative changes) in the heart of control diabetic mice not received camel milk or glibenclamide drug (c)

**FIGURE 6:**
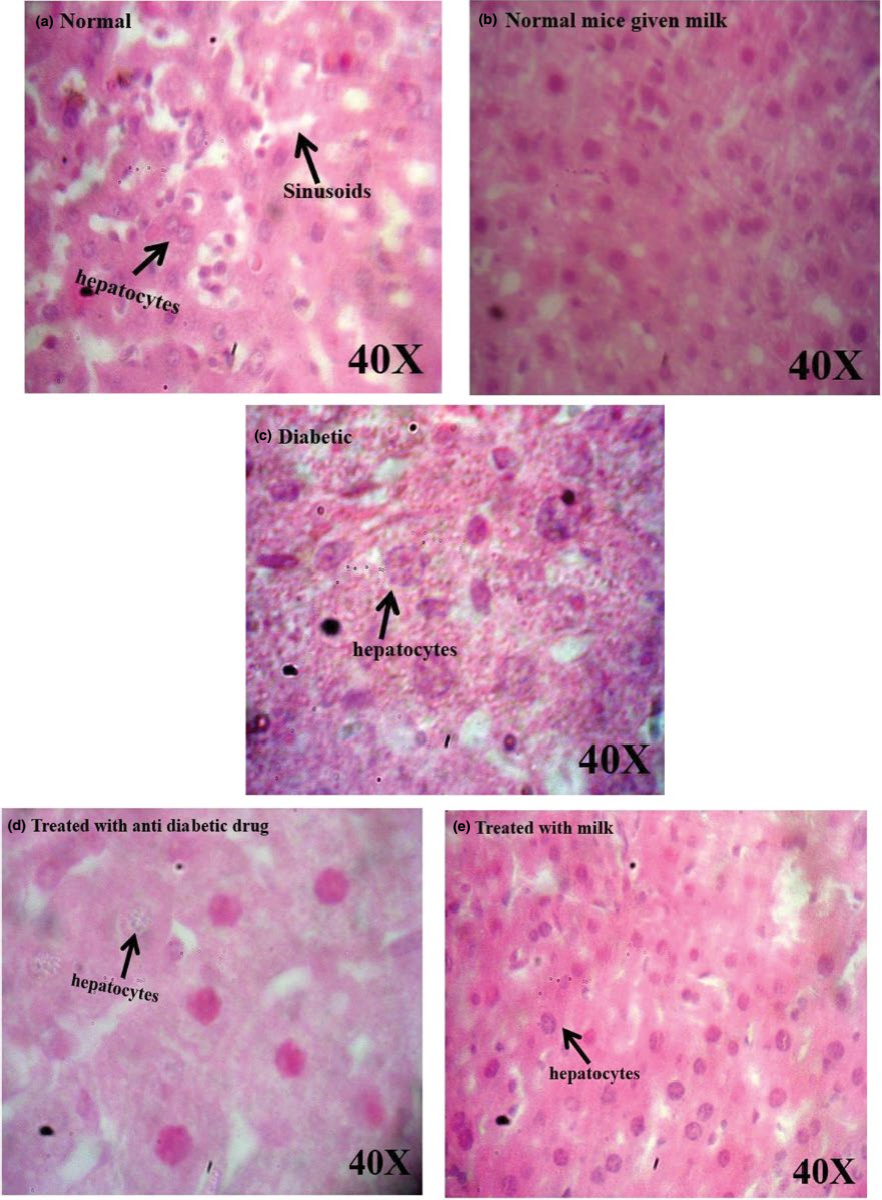
Histopathological evaluation of liver by using H & E staining in different groups of mice. As the figure shows, the pathological changes in the liver of diabetic mice received glibenclamide drug (d) and camel milk (e) were approximately comparable and considerably less than the pathological changes (degenerative changes and hypertrophic hepatocytes) observed on the liver of control diabetic mice (c)

Streptozotocin specifically affects the pancreatic insulin‐secreting β‐cells and induces the increase in the reactive oxygen species (ROS) level in the pancreas, liver, and relative tissues. Increasing ROS levels cause tissue damages and increased lipid peroxidation, which results in the production of free radicals; as a result, the oxidative damage of polyunsaturated fatty acids occurs. In physiological conditions, the tissue concentration of lipid peroxides is low (Gayathri & Kannabiran, 2010). Bolkent et al. (2006) demonstrated that the plasma concentration of lipid peroxides was elevated in diabetic rats. Increased plasma concentration of lipid peroxides in diabetic rats and lipid peroxide‐mediated damage is regarded as one of the characteristic features of chronic diabetes (Bolkent et al., 2006). Tissue damage, due to lipid peroxides, can contribute to the development of both type 1 and 2 diabetes (Lenzen, 2008). The intensity of pathological changes was reached to a minimum in diabetic mice which received camel milk or glibenclamide. Also, a typical histological structure of the heart was observed in normal mice, whereas pathological changes, including confused cellular nuclei and degenerative changes, were observed in control diabetic mice. However, the pathological changes were reached to a minimum in diabetic mice treated with antidiabetic drug and camel milk (Figure 5). Liver showed a usual histological structure in normal mice, while hypertrophic hepatocytes along with degenerative changes were observed in the diabetic control mice (Figure 6). The pathological changes in diabetic mice were treated with camel milk or glibenclamide found less compared with the untreated diabetic mice (Figure 6). Overall, the results of histopathological findings were in accordance with the results of blood glucose, HbA1c, ALT, AST, TG, and cholesterol levels show that camel milk decreased the histopathological lesions compared with the control diabetic group, and the pathological changes were comparable with diabetic mice which received glibenclamide.

## 4 CONCLUSION

In this study, the efficacy of camel milk in the treatment of diabetic mice was evaluated and confirmed. Also, the potency of camel milk to restore the activity of hepatocyte enzymes ALT and AST was evaluated. The results demonstrated that camel milk was approximately effective as much as glibenclamide. In addition, camel milk was efficacious nearly as much as glibenclamide to normalize blood TG and cholesterol concentrations. The results of the present study suggest that camel milk, as a natural and safe product, can be used as an alternative treatment regimen in diabetes therapy.

## CONFLICT OF INTEREST

According to the authors, there are no competing interests.

## Data Availability

Data sharing is not applicable to this article as no new data were created or analyzed in this study.
